# Hydroxypropyltrimethyl Ammonium Chloride Chitosan Nanoparticles Coatings for Reinforcement and Concomitant Inhibition of Anionic Water-Sensitive Dyes Migration on Fragile Paper Documents

**DOI:** 10.3390/polym14183717

**Published:** 2022-09-06

**Authors:** Huiping Xing, Jianwei Wang, Ouya Ma, Xiaolian Chao, Yajun Zhou, Yuhu Li, Zhihui Jia

**Affiliations:** School of Materials Science and Engineering, Engineering Research Center of Historical Cultural Heritage Conservation, Ministry of Education, Shaanxi Normal University, Xi’an 710119, China

**Keywords:** reinforcement, anionic water-sensitive dyes migration, fragile paper

## Abstract

The fragile paper is treated to improve the stability and appearance of the paper artifact, such as washing, lining, deacidification, and reinforcement. During the above treatments, paper documents inevitably make contact with water directly, leading to the appearance change, stability decrease, and migration or fading of anionic water-sensitive dyes, which are seriously harmful to information security. Herein, Hydroxypropyltrimethyl ammonium chloride chitosan (HACC) nanoparticles were employed for the reinforcement and concomitant inhibition of anionic water-sensitive dye migration on fragile paper. HACC nanoparticles were prepared through physical ball grinding method and characterized via LPSA, SEM, TEM, XRD and FTIR. To evaluate the protective potential of HACC nanoparticles coating, the chemical and mechanical properties of coated and uncoated papers were evaluated after dry heat and hygrothermal accelerated aging. Additionally, good color stability of anionic water-sensitive dyes was observed on the paper coated with HACC nanoparticles after lining technology. Finally, the interaction mechanism between the anionic water-sensitive dyes and HACC nanoparticles was analyzed using an ultraviolet spectrophotometer and FTIR. The as-proposed technique can provide technical support to improve the mechanical properties of fragile paper and enhance the anionic water-sensitive dyes stability in the aqueous phase.

## 1. Introduction

**Figure 1:**
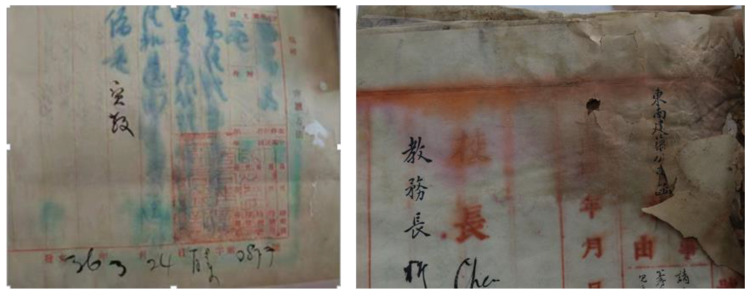
Typical example of blue ink (**left**) and red ink (**right**) migration of 19th century documents.

Paper documents, as one of the major modes of information exchange, are indispensable in transmitting cultural achievements around the world. In particular, archives that record information or images through anionic water-sensitive dyes such as red ink and blue ink have played an important role in Chinese archives before and after the establishment of PRC. Owing to the storage environment, anthropic factors, and microorganisms, the paper and the associated information are subjected to irreversible degradation and loss of valuable information [1,2]. The primary constituent of paper is cellulose, which is easily subjected to the acid hydrolysis of glycosidic bonds and oxidation. Due to the effect of acidity, temperature, and moisture content, the β (1,4)-glycosidic bonds of the cellulose can break via autocatalytic hydrolysis reaction, causing paper embrittlement on the macroscopic scale [3,4,5]. Oxidation is often synergistic with acidification, mainly promoted by acidic medium during the interaction between cellulose, light, or metal ions, leading the paper to turn yellow [6,7]. Deacidification and reinforcement are commonly used for the conservation of cellulose artistic substrates. Considering the primary role of acid-catalyzed hydrolysis, neutralizing the acids present on the surface or the interior of paper is the main chemical strategy to delay the degradation of paper documents. Owing to relatively good alkaline reserves, low toxicity, moderate price, and excellent deacidification performance, aqueous inorganic alkaline solutions of calcium, magnesium, and barium have been widely used for several decades as compared with the organic base [8,9,10,11]. However, the use of aqueous alkaline solutions has often led to undesirable side effects, such as drastic modifications of the appearance of paper, mechanical damage of brittle papers, and even the inevitable diffusion of water-sensitive dye images, seriously damaging the original archive information [12,13,14,15]. Lining technology is the most widely used method in paper archives conservation in China [16]. The aged paper inevitably comes in direct contact with water during various processes in lining technology, such as washing, deacidification, and wet support, leading to the migration of water-sensitive dye and pulp of fragile paper, which causes serious harm to the security of documents (Figure 1). Herein, a new method was proposed that can reinforce the fragile paper without risk of loss of the image color, and at the same time, maintain the original hydrophilic properties of the fragile paper for the successful implementation of traditional lining.

**Figure 2:**
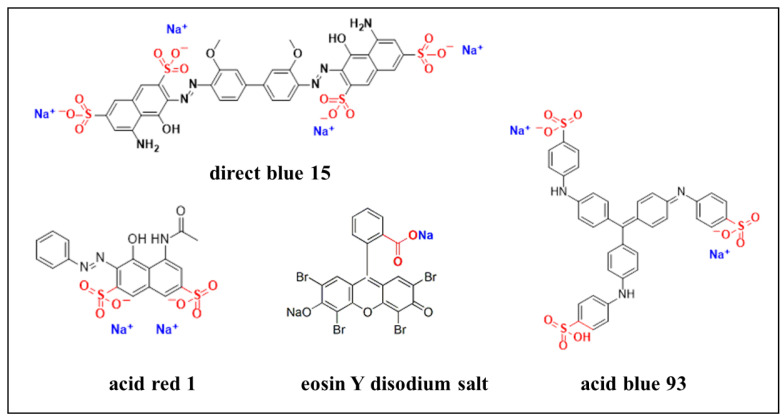
Molecular structure diagram of water-soluble anionic ink dyes.

Anionic water-sensitive dyes such as direct blue 15, acid blue 93, acid red 1, and eosin Y disodium salt are the main components of blue and red inks (Figure 2). Due to poor light, water, acid, and alkali resistance of the dye [17], the water-sensitive dye ink in the paper file handwriting has low durability, and it migrates upon interaction with water. Using the cationic fixatives in aqueous solution treatment is effective in preventing the migration of anionic dyes [18]. Therefore, it is possible to use cationic fixatives to prevent the migration of anionic water-sensitive dyes in lining technology on paper treatment, and play a role in strengthening the fragile paper. Chitosan (CS) is used in various applications due to its special advantages such as biocompatibility, non-toxicity, and biodegradability [19]. As a derivative of chitosan, Hydroxypropyltrimethyl ammonium chloride chitosan (HACC) is an excellent cationic polymeric flocculant, has shown great adsorption performance towards some anionic dyes, and can form stable chelates with most transition metal ions [20]. With good film-forming performance, antibacterial, and bacteriostatic properties and high positive charge density, HACC has potential applications in food industry, papermaking, medicine, industrial water treatment, textile processing, and other fields [21]. Due to high reactivity, dispersion of nanoparticles has been proposed as an efficient treatment of paper [22,23,24,25,26,27,28,29]. 

Herein, HACC nanoparticles have been studied for the reinforcement and concomitant inhibition of water-sensitive dye migration on fragile paper in this work. HACC nanoparticles were prepared through physical ball grinding method and characterized via laser particle size analyzer (LPSA), scanning electron microscopy (SEM), transmission electron microscopy (TEM), X-ray diffraction (XRD), and Fourier transform infrared spectroscopy (FTIR). The effects of HACC on the paper and water-sensitive dye images were studied. To evaluate the resistance of chitosan nanoparticles coating, comparative experiments were performed between the uncoated paper and the ones coated with HACC nanoparticles. The degradation of mechanical properties of paper after artificial aging was evaluated via tensile strength measurements of samples after dry heat and hygrothermal accelerated aging. On the other hand, red ink and blue ink were used to study the color stability of anionic water-sensitive dyes on the paper coated by the HACC nanoparticles via immersion measurements. Furthermore, the probable interaction mechanism of anionic water-sensitive dyes and HACC was analyzed using ultraviolet spectrophotometer and FTIR. 

## 2. Materials and Methods

### 2.1. Materials

All the reagents used were of analytical grade. Hydroxypropyltrimethyl ammonium chloride chitosan (HACC, Purity ≥ 95%) served as the basic reagent for the experiments and was purchased from Zhejiang Aoxing Biotechnology Co. LTD. Isopropanol was purchased from Sinopharm Group Chemical Reagent Co. LTD. The Xuan paper (density 30 g/m^2^) of Jingxian Purple Light Paper Industry, Anhui Province, was used as the paper sample. 

### 2.2. Particles Preparation and Characterization

A physical ball grinding method was adopted to prepare HACC nanoparticles due to its convenience and simple operation [30]. HACC was dried for 4 h at 60 °C and placed into ball mill tank containing 5 mm diameter zirconia grinding balls. The QM-3SP2-CL planetary ball mill was employed at 401 rpm speed for 24 h. The HACC of submicron particle size was obtained by separating zirconia pellets, and then, drying was performed at 60 °C for 4 h. The resultant was poured into a ball grinding tank, and ball grinding was continued for 24, 48, 72, 96, and 120 h. 

Laser Particle Size Analyzer. The Bi-90plus laser particle size analyzer was used to measure the particle size of HACC. HACC after ball grinding was ultrasonically dispersed in anhydrous ethanol for 20 min in the concentration of 0.01–0.1% (*v*/*v*). 

Scanning Electron Microscopy (SEM). The morphology of HACC particles and specimens before and after treatment were observed under a scanning electron microscope (FEI, Quanta 200) and a comparative analysis was conducted. A silicon wafer was used as the base surface and was tested after gold spray treatment. Analyses were performed at low vacuum (1–150 Pa) at the accelerating voltage of 5 kV and magnification of 6000× and 100,000×.

Transmission Electron Microscopy (TEM). The microstructure of HACC was observed under Tecnai G2 F20 type field emission transmission electron microscope with a maximum resolution of 2.3 Å and equipped with a LAB-6 cathode and a CCD camera (Olympus). The HACC prepared via ball grinding for 120 h was added to isopropyl alcohol for ultrasonic dispersion for 20 min, and then, placed into a carbon TEM grid for imaging. 

Fourier Transform Infrared Spectroscopy (FTIR). Fourier transform infrared analysis of HACC before and after ball grinding were carried out using Thermo Nicolet IS10 Spectrometer in the range of 4000 and 400 cm^−1^ with KBr pellet method. The number of scans was 16, and the resolution was 4 cm^−1^.

X-ray Diffraction (XRD). The phase composition and crystallinity of chitosan before and after ball grinding were analyzed via DX-2700 X-ray diffractometer (Dandong Haoyuan Instrument Co., Ltd., Dandong, China) with Cu Kα radiation under the voltage of 40.0 kV and current of 15.0 mA at room temperature. The XRD patterns were recorded at the scan rate of 2°/min.

### 2.3. Particles Application on Model Paper

Xuan paper, a Chinese hand-made paper, was selected for the experiments because it has traditionally been used for Chinese paintings as well as calligraphy, and is widely used in paper conservation. The specimen notations for different treatments are as follows. The specimen was treated with isopropanol solution containing HACC (equivalent weight HACC nanoparticles dispersed in 1 L of isopropanol solution under ultrasonic conditions for 30 min). Then, 30 mL of dispersive solutions of HACC of different concentrations were sprayed on a 35 × 35 cm Xuan paper, and the samples were placed into the BCM-1000 bio-cleaning worktable and the isopropanol were completely volatilized under low wind speed. Before testing, the samples were kept at (23 ± 1) °C with the relative humidity of (50 ± 2) % for 24 h according to GB/T 10739-2002.

Contact Angle Test. The hydrophilicity of the paper samples was characterized by the contact angle test. The OCA20 video optical contact angle tester was used, and the droplets were deionized water. The untreated and HACC-treated paper samples were cut into 3 cm × 3 cm and placed flat. All the samples were measured at five positions, and the average value was calculated with standard deviation. 

Accelerated Aging Test. The artificial accelerated aging test was performed in a BH0-402A aging chamber at 105 °C for 72 h as per ISO 5630-1:1991. The hygrothermal accelerated aging of the specimens was conducted at 80 °C and 65% relative humidity (RH) for 72 h according to GB/T 22894-2008. The tensile strength of the specimens was measured with QT-1136PC universal material testing machine with 270 mm × 15 mm according to ISO 1924-2:1994 method. The folding endurance of the specimens of 150 mm × 15 mm was measured with the LB-MIT135 tester according to ISO 5626:1993 method. The tearing strength of the specimens was measured with LB-SL1000 tester with (63 ± 0.5) mm × (50 ± 2) mm according to GB/T 455-2002. The samples were cut according to the machine (MD) and cross directions (CD) of paper. 

### 2.4. Fixation of Anionic Water-Sensitive Ink Dyes

Blue ink and red ink were applied on the paper using a brush and placed at ambient temperature until dry. Partial samples were sprayed with 3 mL HACC nanoparticles dispersion on double sides, while the others were not. After 1 week at constant room humidity (RH 65%, 25 °C), the samples were sprayed with water and the migration of anionic water-sensitive dyes was observed via optical microscopy. 

### 2.5. Adsorption Mechanism of HACC to Water-Sensitive Ink Dyes

**Figure 3:**
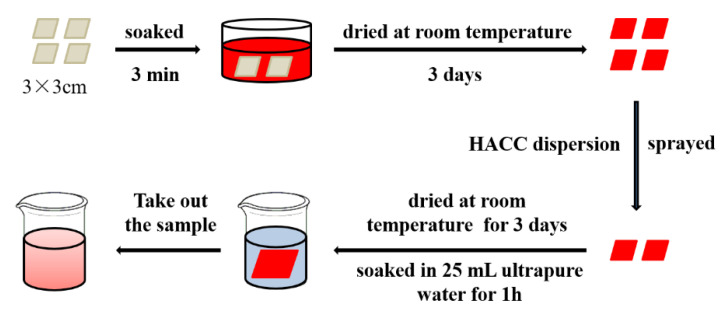
The flow chart of immersion experiment.

Direct blue 15, acid blue 93, acid red 1, and eosin Y disodium salt were prepared into a 2.5 g/L solution. The paper samples of 3 mm × 3 cm size were soaked in the above prepared dye solutions for 3 min, and then dried at room temperature. Part of the samples was sprayed with HACC nanoparticles dispersion double sides and placed at room temperature for 3 days. The treated and untreated samples were soaked in 25 mL ultrapure water for 1 h, and then taken out. The absorbance spectrum of the desorbed dye solution was recorded using a Shimadzu-2600 UV-Vis spectrophotometer (Figure 3).

A pipet-gun was used to measure 15 mL of 5 g/L aqueous solutions of Direct blue 15, acid blue 93, acid red 1, and Eosin Y disodium salt, respectively, into four beakers. HACC nanoparticles were added to each beaker and stirred at room temperature for 20 min. Part of the obtained samples solution was diluted 20 times and then observe the phenomenon. Another part of the samples was filtered and dried in a blast drying oven at 40 °C. A Nicolet iS10 FT-IR spectrometer coupled with a diamond ATR module was used in the reflection mode at room temperature and ambient humidity. The wave numbers ranged from 500 to 4000 cm^−1^ with a resolution of 4 cm^−1^, and the scan number was 64 times.

## 3. Results and Discussion

### 3.1. Characterization of HACC Nanoparticles

**Figure 4:**
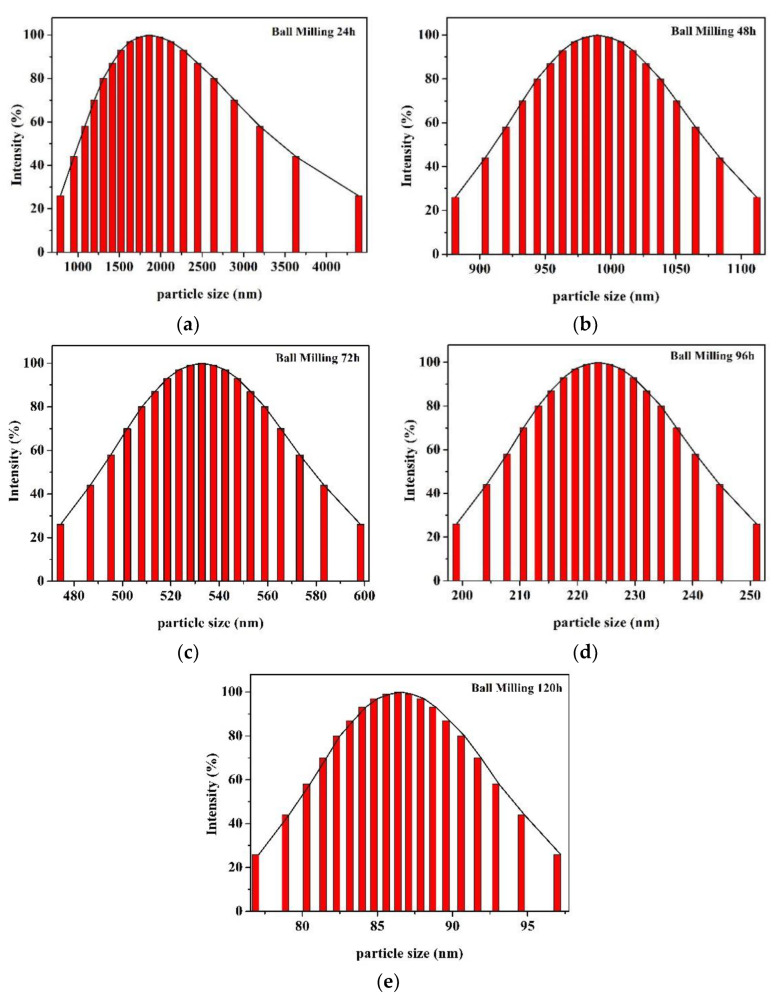
Size distributions of HACC obtained from ball milling: (**a**) 24 h ball milling; (**b**) 48 h ball milling; (**c**) 72 h ball milling; (**d**) 96 h ball milling; and (**e**) 120 h ball milling.

The HACC particles were characterized via LPSA, SEM, TEM, XRD, and FTIR. Figure 4 shows the size distribution of HACC after ball milling for 24 h, 48 h, 72 h, 96 h, and 120 h. As the ball grinding time increased, the size of HACC particles was gradually reduced. The average particle size of HACC samples after ball grinding for 120 h was 86.6 nm. 

**Figure 5:**
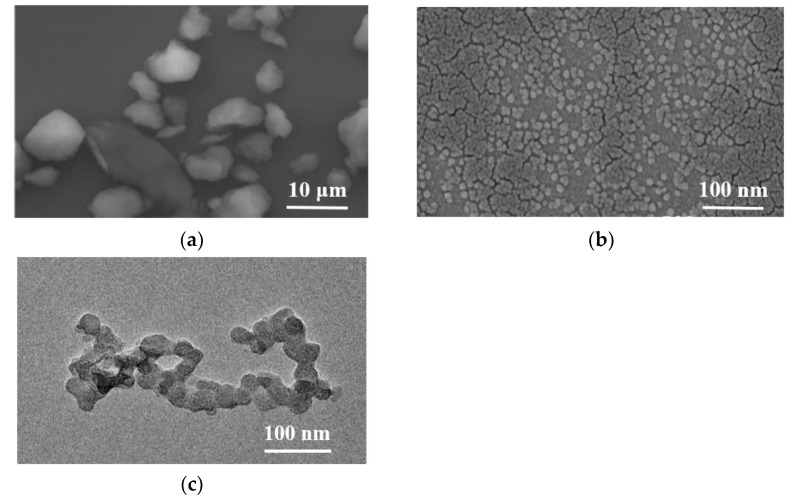
SEM images of (**a**) original HACC (6000×) and (**b**) 120 h ball milling (100,000×); (**c**) TEM images of HACC after ball milling for 120 h.

Figure 5a,b present the SEM images of original HACC particles and HACC particles after ball milling for 120 h. There were significant differences between the particle sizes of both. The particle size distribution of HACC without ball grinding was extremely uneven, and the average particle size was larger than 5 microns. The HACC nanoparticles was obtained after ball grinding for 120 h. The particle size was evenly distributed with average size of 20–100 nm, which is consistent with the TEM results (Figure 4e). Compared with Figure 5c, the average particle size of HACC was bigger. This is primarily because the HACC nanoparticles underwent easy crosslinking while using laser particle size instrument testing. Additionally, concentration has remarkable effects on the size of the nanoparticles [23]. The results show that the HACC nanoparticles were successfully prepared by ball milling for 120 h.

**Figure 6:**
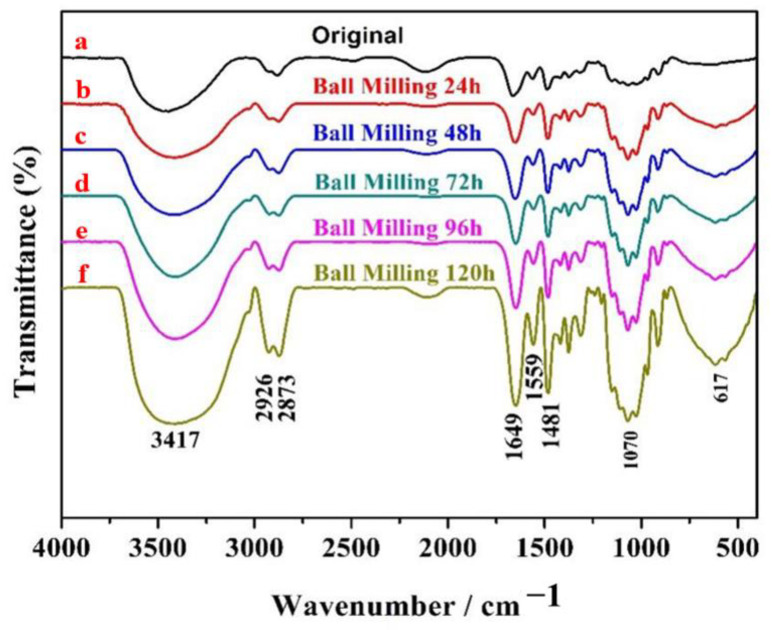
FT-IR spectra of HACC milled for different times: (**a**) HACC; (**b**) ball milling 24 h; (**c**) ball milling 48 h; (**d**) ball milling 72 h; (**e**) ball milling 96 h; and (**f**) ball milling 120 h.

Figure 6 presents FT-IR spectra of HACC milled for different times. As presented in Figure 6a, the stretching vibration superposition peaks of -OH and -NH were observed at 3417 cm^−1^. There were weak spectral bands at 2926 cm^−1^ and 2873 cm^−1^, which were caused by the stretching of methyl and methylene CH moieties in the molecule. Characteristic peaks of N-H stretching vibration peaks appeared at 1649 cm^−1^ and 1559 cm^−1^. First, 1481 cm^−1^ presents the bending vibration peak of C-H bond in trimethyl quaternary ammonium ion, while 1150 cm^−1^, 1070 cm^−1^, and 1027 cm^−1^ display the characteristic peak of C-O stretching vibration [31,32,33]. The main absorption peaks of HACC remained unchanged after ball milling for 24 h, 48 h, 72 h, 96 h, and 120 h. These results indicate that the molecular size of HACC was reduced by ball grinding without destroying its original structure.

**Figure 7:**
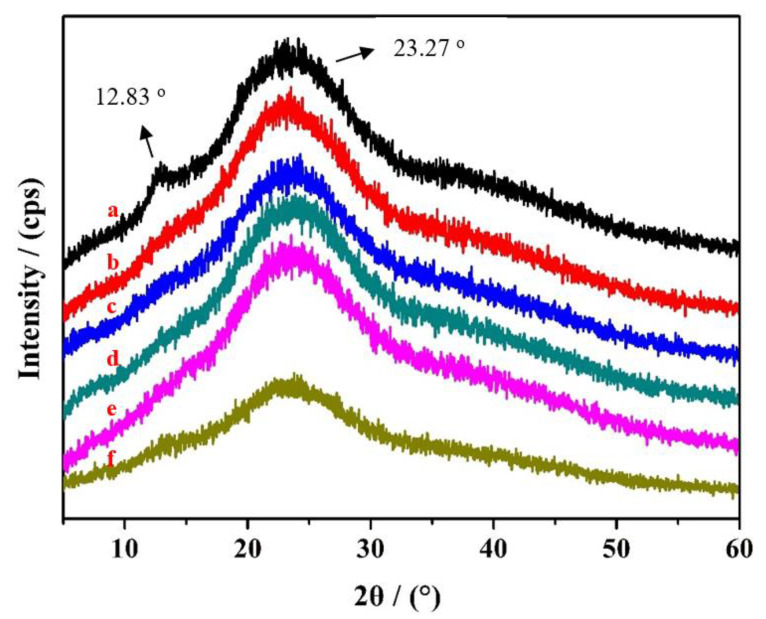
X-ray diffraction patterns of HACC milled for different times: (**a**) original HACC; (**b**) 24 h ball milling; (**c**) 48 h ball milling; (**d**) 72 h ball milling; (**e**) 96 h ball milling; and (**f**) 120 h ball milling.

XRD patterns of the HACC milled for different times were obtained, and the corresponding results are presented in Figure 7. As shown in Figure 7a, two intense characteristic peaks occurred at 2θ of 12.83° and 23.27° [34]. The characteristic bands at 2θ of 12.83° decreased with ball milling (Figure 7a–e), while the characteristic bands at 2θ of 23.27° displayed no prominent change. Only one characteristic peak was observed when ball grinding was performed for 120 h, and the diffraction peak was significantly weakened (Figure 7f). This is because the intermolecular hydrogen bonds of HACC were destroyed by ball grinding and the crystallinity of HACC was decreased. 

### 3.2. Conservation Applications of HACC Nanoparticles

**Figure 8:**
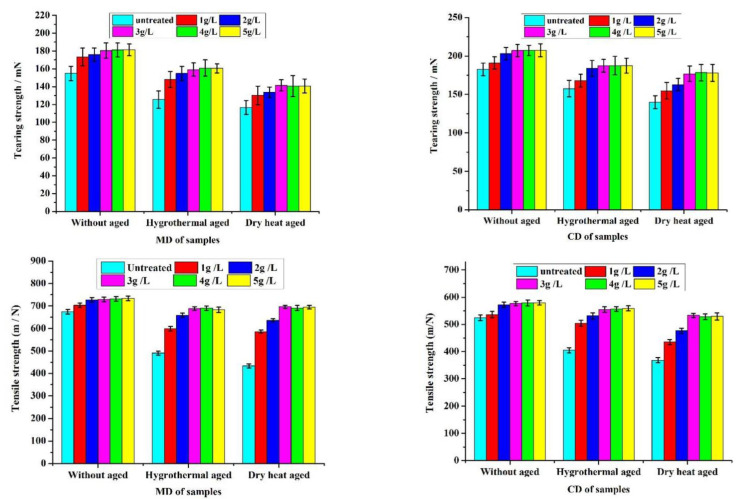
Tearing strength and tensile strength of the uncoated and coated paper before and after accelerated aging tests.

Temperature and moisture are vital factors in paper degradation. Herein, dry heat aging and hygrothermal aging were adopted to evaluate the resistance of the coated paper. In general, the tearing strength and tensile strength are important indicators that reflect the cellulosic structure and suitability of the paper [35]. The tearing strength and tensile strength for the uncoated paper and coated with chitosan nanoparticles before and after accelerated aging tests are shown in Figure 8. Both the tearing strength and tensile strength of the coated paper increased with the increase in concentration of HACC nanoparticles as compared to uncoated paper. These results indicate that HACC nanoparticles may play an important role in improving the mechanical properties of the paper. When the concentration of HACC nanoparticles was greater than 3 g/L, there was no prominent improvement in the mechanical properties. Therefore, the concentration of HACC nanoparticles was taken as 3 g/L in the subsequent studies. There was a significant difference between the MD and CD of the paper with regard to the tearing strength of the paper; generally, MD was greater than CD with regard to tensile strength. The coated papers displayed better thermal and wet stability and clearly exhibited a lower loss of paper strength than the uncoated papers. The declining trend of dry heat aging durability was more prominent than that of hygrothermal aging. Compared with the uncoated paper, the paper treated with HACC nanoparticles demonstrated superior durability.

**Figure 9:**
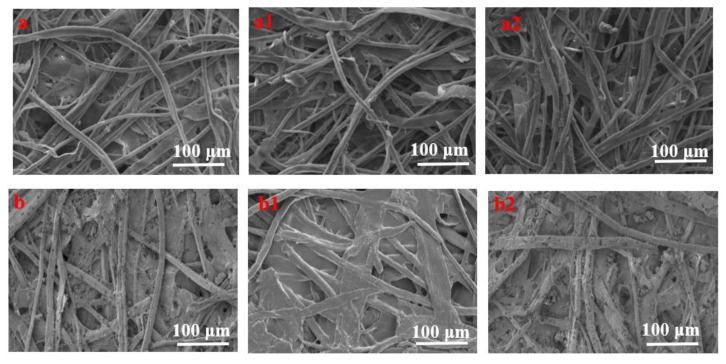
SEM micrographs of Xuan paper specimens: (**a**) uncoated paper; (**a1**) uncoated paper after hygrothermal aging; (**a2**) uncoated paper after dry heat aging; (**b**) coated with HACC nanoparticles; (**b1**) coated paper after hygrothermal aging; and (**b2**) coated paper after dry heat aging.

Figure 9 presents the SEM images of Xuan paper samples that were uncoated and coated with HACC nanoparticles before and after accelerated aging tests. Figure 9a–a2 show the surface morphology of the uncoated Xuan paper sample before and after accelerated aging. There were wide pores between the network of entwined cellulose fibers; however, after dry heat aging and hygrothermal aging, the fibers became locally ruptured and underwent different degrees of fracture. Compared to the untreated samples, HACC nanoparticles were dispersed on the surface of the cellulose fibers and filled the pores in the internal fiber mesh to form a protective layer (Figure 9b). After hygrothermal aging, HACC demonstrated a membrane phenomenon. This is because HACC nanoparticles have good solubility in water and excellent film-forming property. The HACC nanoparticles were distributed in the void of paper fiber in the granular form, which upon treating with water, dissolved to form a film covering the paper fiber. The cellulose molecules have high density of hydroxyl groups and can be easily adsorbed on the cellulose by van der Waals force and hydrogen bonding interactions. After dry heat aging, the HACC nanoparticles remained granular and dispersed on the surface of the paper and in the cracks between the paper fibers. Combined with Figure 8, the wrapped and compact fiber can prevent possible degradation due to temperature and humidity.

**Figure 10:**
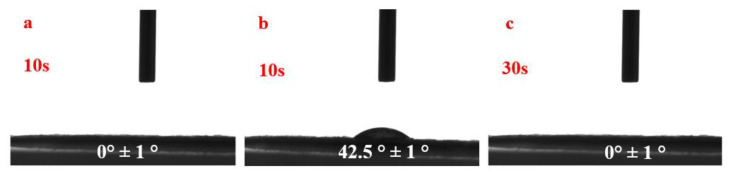
Contact angle of Xuan paper samples before and after coated with HACC nanoparticles: (**a**) uncoated paper after 10 s; (**b**) coated paper after 10 s; and (**c**) coated paper after 30 s.

Contact angles for pure water were obtained to evaluate the hydrophilic-hydrophobic balance of the coated paper. As shown in Figure 10, untreated paper was hydrophilic and had high wettability (Figure 10a). After treatment with HACC nanoparticles, the coated slide displayed slight hydrophobic character with a contact angle of ∼42.5°. Notably, the contact angle dropped to 0° after 30 s. Combined with Figure 10b, the main reason for the phenomenon is that HACC nanoparticles filling the space between the paper fibers dissolved gradually over time causing the increase in hydrophilicity of the paper. This is consistent with more hydrophilic character and enormous surface area of nanoscale structures [36]. Hence, the paper treated with HACC nanoparticles remained hydrophilic and did not affect the subsequent aqueous conservation treatments. 

### 3.3. Fixation of Anionic Water-Sensitive Dyes

**Figure 11:**
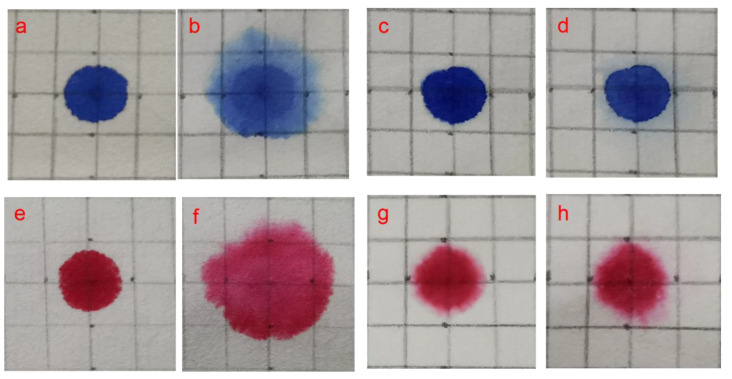
Fixation of blue ink and red ink on natural aged filter paper ((**a**,**e**): untreated; (**b**,**f**): untreated samples after sprayed with water; (**c**,**g**): treated with HACC nanoparticles on both sides; and (**d**,**h**): treated samples after sprayed with water).

Red ink and blue ink were used to study the color stability of anionic water-sensitive dyes on the treated and untreated paper through immersion measurements (Figure 11). A comparison of photomicrographs taken before and after fixing with HACC nanoparticles and after wetting provides an indication of the amount of bleeding/fading that occurred in the ink. After fixing with HACC nanoparticles, the red and blue ink dots retained their original appearance (Figure 11c,g). After being sprayed with water, the untreated red and blue ink dots clearly bled and faded (Figure 11b,f) in less than a minute. The treated blue ink dots displayed slight alterations such as small halos or migration to the verso side, while the treated red ink dots withstood the aqueous treatment unchanged and retained their original appearance. 

**Figure 12:**
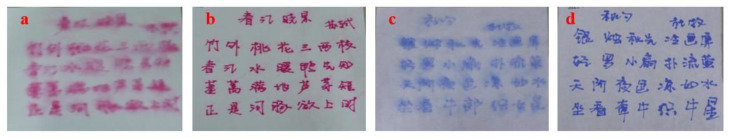
Pictures of paper with red and blue ink handwriting after lining technology: (**a**,**c**): untreated; and (**b**,**d**): treated with HACC nanoparticles.

Pictures of paper with red and blue ink handwriting after lining technology were shown in Figure 12. It can be seen that the writing is blurred by migration before treated with HACC nanoparticles (Figure 12a,c). The paper samples treated with HACC nanoparticles come in direct contact with water during various processes in the traditional Chinese lining technology without any risk of loss of color (Figure 12b,d).

### 3.4. Adsorption Mechanism of HACC to Water-Sensitive Ink Dyes

**Figure 13:**
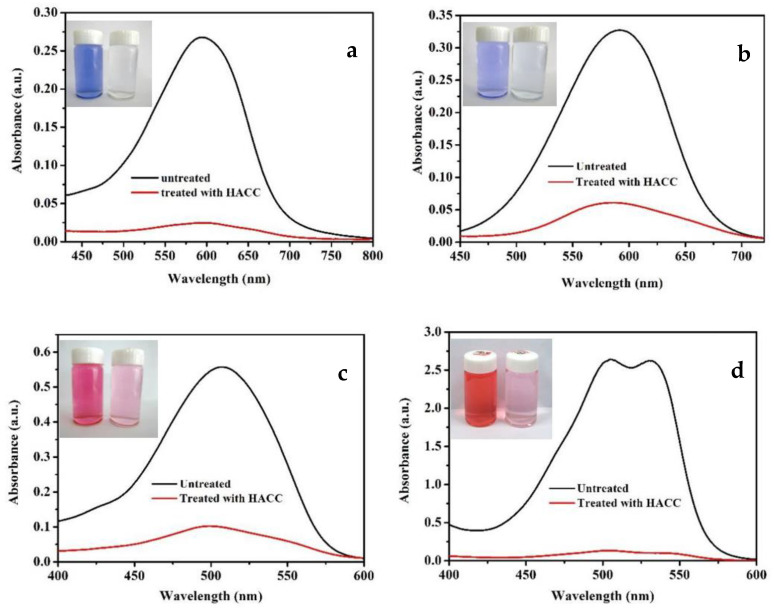
UV-Vis absorption spectra of dye solution before and after treatment with HACC nanoparticles ((**a**): Direct blue 15; (**b**): Acid blue 93; (**c**): Acid Red 1; and (**d**): Eosin Y disodium salt).

The adsorption of HACC nanoparticles on the Direct blue 15, acid blue 93, acid red 1 and Eosin Y disodium salt can be verified via immersion experiments. Figure 13 presents the UV-Vis absorption spectra of the dye solution before and after treatment with HACC nanoparticles. The HACC nanoparticles displayed adsorption effect towards all kinds of dyes, especially, on the Direct blue 15, acid ink blue, and blue ink. 

**Figure 14:**
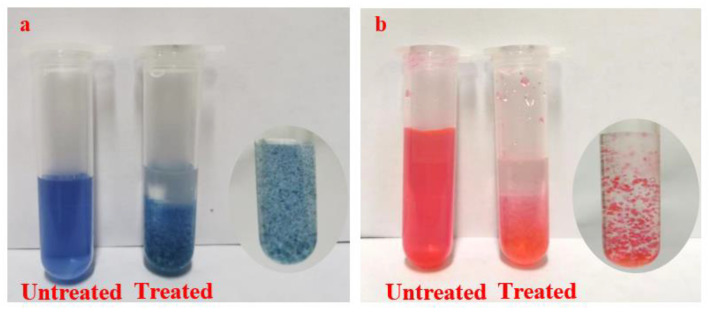
Picture of dye solution before and after treatment with HACC nanoparticles ((**a**): Direct blue 15; (**b**): Acid Red 1).

Figure 14 shows the picture of dye solutions before and after treatment with HACC nanoparticles. It can be seen that dye solutions after treatment with HACC nanoparticles formed gelatinous particles, indicating the adsorption of dye and HACC nanoparticles.

**Figure 15:**
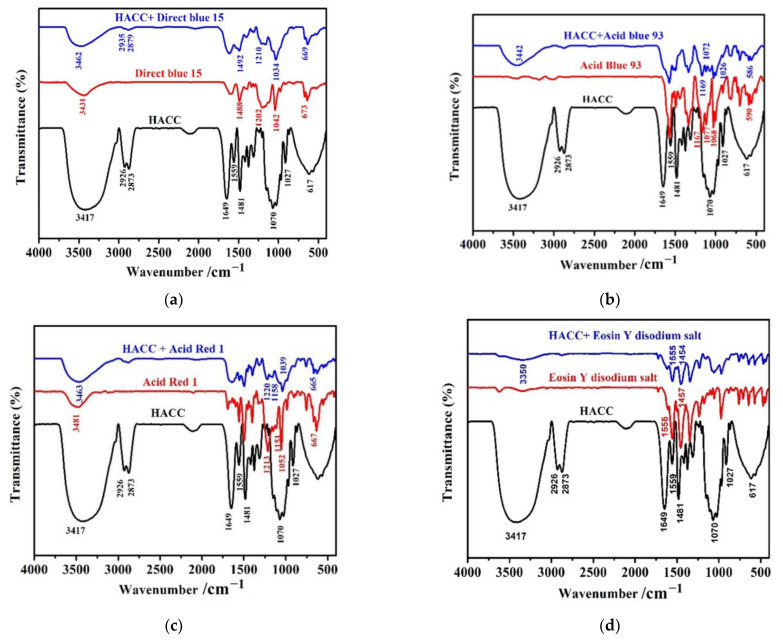
Infrared spectra of HACC nanoparticles before and after adsorption ((**a**): Direct blue 15; (**b**): Acid blue 93; (**c**): Acid red 1; and (**d**): Eosin Y disodium salt).

The FTIR spectra of HACC nanoparticles, direct blue 15, and acid blue 93 samples after HACC adsorption are shown in Figure 15a. The characteristic peaks of HACC nanoparticles were also found in the HACC nanoparticles samples after direct blue 15 adsorption. The OH/NH stretching vibration clearly broadened and shifted from 3431 cm^−1^ to 3462 cm^−1^. The stretching of the methyl and methylene CH moieties at 2926 cm^−1^ and 2873 cm^−1^ were shifted to higher wavenumbers after adsorption. The characteristic peaks of direct blue 15 at 1488 cm^−1^ and 1202 cm^−1^ corresponding to the bending vibration peak of C-H bond were shifted to higher wavenumbers, while the C-O stretching vibration at 1042 cm^−1^ was shifted to a lower wavenumber. This phenomenon can be attributed to the chemical interaction between the HACC nanoparticles and direct blue 15. The peak at 673 cm^−1^ shifted to a lower wavenumber after interaction with HACC nanoparticles. This phenomenon can be attributed to the SO^3−^ of direct blue 15, implying the participation of SO^3−^ in the adsorption progress. Similar peak changes were observed in the infrared spectra of acid blue 93, acid red 1, and eosin Y disodium salt after adsorption with HACC nanoparticles (Figure 15b–d). 

**Figure 16:**
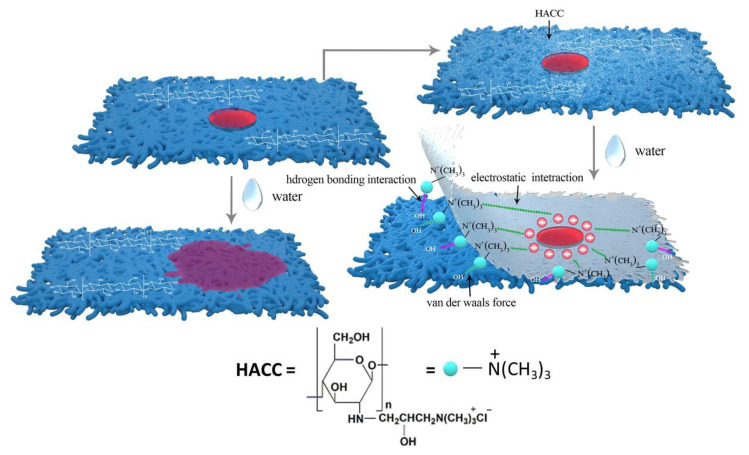
Schematic drawing for possible interactions between HACC nanoparticles and cellulose and dye molecules.

Based on the above discussion, a schematic drawing for the possible interactions between the HACC nanoparticles and cellulose and dye molecules is presented in Figure 16, which primarily includes the following two aspects: (1) interaction with paper cellulose: the cellulose molecules have high density of hydroxyl groups and can be easily adsorbed on the cellulose-based bioadsorbents by van der Waals force and hydrogen bonding interactions [37]. The HACC nanoparticles were distributed in the void of paper fiber in the granular form, and it dissolved in water and formed numerous hydrogen bonds with the paper fiber. (2) Interaction with dye: The HACC nanoparticles, acting as cationic fixatives, could firmly adsorb the anionic dyestuffs on the fiber via electrostatic adsorption and form an insoluble precipitate, which prevented the dye from migrating in water. Notably, the treated paper kept the paper hydrophilic so that the subsequent water conservation treatment could be conducted. 

## 4. Conclusions

In conclusion, HACC nanoparticles, acting as cationic fixatives, were applied to prevent water-sensitive dye images from migrating in water, and at the same time, had a reinforcement function for aged paper documents. HACC nanoparticles were prepared through a physical ball grinding method and characterized via LPSA, SEM, TEM, XRD, and FTIR. The results show that the HACC nanoparticles with a uniform particle size distribution of 20–100 nm were successfully prepared by ball milling for 120 h. The basic structure of HACC did not change after ball grinding. Specifically, the branch chain of HACC was basically not destroyed. The water-sensitive dye on the treated paper was well-protected from migration due to aqueous cleaning and deacidification or applying water-containing adhesives. Compared to the untreated samples, HACC nanoparticles were dispersed on the surface of the cellulose fibers and filled the pores in the internal fiber mesh to form a protective layer. The resistance of coating paper was evaluated by comparative experiments through artificial aging. The results show that the coated paper with HACC nanoparticles displayed superior durability with tensile strength and tearing strength due to its good solubility in water and excellent film-forming property. Additionally, the original hydrophilicity of paper was maintained, which is very advantageous to reinforce the fragile paper before it is exposed to water in the lining technology. Finally, the mechanism of interaction between the HACC nanoparticles and paper and water-sensitive dye image was analyzed using ultraviolet spectrophotometer and FTIR. The HACC nanoparticles displayed adsorption effect towards all kinds of dyes through forming gelatinous particles, especially on the direct blue 15, acid ink blue, and blue ink. The as-proposed process is easy, does not need expensive equipment, and is free from hazardous solvents. In the future, it is possible to effectively save on manpower and material resources and prevent damage to paper in the repair process.

## Data Availability

Not applicable.
